# Toward Precision Healthcare: Context and Mathematical Challenges

**DOI:** 10.3389/fphys.2017.00136

**Published:** 2017-03-21

**Authors:** Caroline Colijn, Nick Jones, Iain G. Johnston, Sophia Yaliraki, Mauricio Barahona

**Affiliations:** ^1^Department of Mathematics, Imperial College LondonLondon, UK; ^2^EPSRC Centre for Mathematics of Precision Healthcare, Imperial College LondonLondon, UK; ^3^School of Biosciences, University of BirminghamBirmingham, UK; ^4^Department of Chemistry, Imperial College LondonLondon, UK

**Keywords:** precision medicine, precision healthcare, data science, precision public health, mathematical modeling

## Abstract

Precision medicine refers to the idea of delivering the right treatment to the right patient at the right time, usually with a focus on a data-centered approach to this task. In this perspective piece, we use the term “precision healthcare” to describe the development of precision approaches that bridge from the individual to the population, taking advantage of individual-level data, but also taking the social context into account. These problems give rise to a broad spectrum of technical, scientific, policy, ethical and social challenges, and new mathematical techniques will be required to meet them. To ensure that the science underpinning “precision” is robust, interpretable and well-suited to meet the policy, ethical and social questions that such approaches raise, the mathematical methods for data analysis should be transparent, robust, and able to adapt to errors and uncertainties. In particular, precision methodologies should capture the complexity of data, yet produce tractable descriptions at the relevant resolution while preserving intelligibility and traceability, so that they can be used by practitioners to aid decision-making. Through several case studies in this domain of precision healthcare, we argue that this vision requires the development of new mathematical frameworks, both in modeling and in data analysis and interpretation.

## Introduction: precision medicine and its challenges

The phrase “precision medicine” describes the idea of delivering the right treatment to the right person at the right time. Precision approaches aim to achieve a medical revolution: individualized therapies based on quantitative, patient-specific datasets, integrated via algorithmic analyses that can aid patient stratification, monitoring, and treatment design. These approaches have been broadly supported in the US under President Obama's Precision Medicine Initiative (Collins and Varmus, 2015; White House Precision Medicine Initiative, 2016), by the Gates Foundation (Cisneros, 2016), and by the Chan Zuckerberg Initiative (Chan Zuckerberg Initiative — Advancing human potential and promoting equal opportunity, 2017). Although some of the aspects of this vision date back to the inception of the Human Genome Project, precision medicine now expands beyond the restrictions of genomics to encompass a wide range of data sources increasingly available to clinicians. The idea of embedding diagnostics and treatment with omics and other medical and physiological datasets at the heart of medicine has been variously described as systems medicine, personalized medicine, computational systems biomedicine, P4 (Predictive, Preventative, Personalized, Participatory) medicine, and precision medicine, to name just a few (Duffy, 2016).

The development of the field has been underpinned by some striking successes, particularly in cancer (Derks et al., 2014; Hiley et al., 2014; Arnedos et al., 2015; Friedman et al., 2015; Navin, 2015; Rubin, 2015; Stover and Wagle, 2015; Wills and Mead, 2015; Cloney, 2017), where molecular profiling is increasingly routine in lung, breast, and colorectal cancers, as well as in leukemias and melanomas (Larry Jameson and Longo, 2015). In asthma, the heterogeneity in clinical response has been shown to overlap with differences in a number of predictive biomarkers, allowing patient stratification for tailored therapies (Muraro et al., 2016). Precision approaches can have immediate benefits for drug repurposing and treatment: the link between type 2 diabetes and early stage Alzheimer's, in which there is often impaired glucose metabolism in the brain, is giving rise to a body of research for new therapeutics that includes repurposing existing drugs (Yarchoan and Arnold, 2014). Cardiovascular disease is another natural domain for precision medicine, as chronic, pervasive problems like diabetes, obesity, and hypertension (with a significant socio-economic and life style component) are directly linked to severe disease including heart failure (Antman and Loscalzo, 2016). These highly prevalent conditions are themselves diverse, multifactorial, and co-occurrent in many individuals, yet mechanism-based markers that predict the development of hypertension can already be identified based on functional genetic and epi-genetic markers (El Shamieh and Visvikis-Siest, 2012; Zhang et al., 2015). In the domain of infectious diseases, precision technologies can also be used to identify pathogens and to determine susceptibility to antimicrobial agents, guiding prescription, e.g., CD4+ cell counts and viral loads can guide HIV therapies (Barnett et al., 2008). Beyond single infections, the function of the microbiome is being probed for disease associations (Gilbert et al., 2016) and metabolomics and integrated omics' tools are revealing disease phenotypes (Chen et al., 2012; Dorrestein et al., 2014).

While there are plenty of potential “low hanging fruits” yet to be plucked, for precision medicine to maximize its impact as envisioned, a number of significant challenges need to be met across multiple domains. Some of these challenges are technical and relate to data collection, processing, storing, and sharing (Garber and Tunis, 2009; Servant et al., 2014; Palmisano et al., 2016; Sboner and Elemento, 2016), and have broad scientific, clinical, social, and ethical ramifications (Juengst et al., 2012; Khoury et al., 2012; Castaneda et al., 2015; Schork, 2015; Cohn et al., 2016). Indeed, advances in sequencing, metabolomics, biomarker discovery, genetics and single-cell technologies, alongside computing, and data science, have brought a strong impetus to the development of the scientific toolkit, data management systems, and regulatory framework for precision medicine. Data collection is currently taking place across the traditional channels of hospitals, community health care settings, and public health bodies, but also increasingly in a decentralized manner via social media analytics and wearable devices. The adoption of systematic formats for Electronic Health Records has improved data collection and consistency, but a considerable effort in data processing and integration still needs to take place (Garber and Tunis, 2009; Servant et al., 2014; Palmisano et al., 2016; Sboner and Elemento, 2016). Storing and accessing extremely high volumes of data is difficult and a concerted effort must be developed to enable clinicians, policy-makers, and academics to access these datasets, thus reducing the need for custom bioinformatics expertise. The question of whether data management is done by public or private organizations, and whether researchers and other users will need to pay to use data is an additional area of concern. Further, efforts to harness large datasets will require the development of sophisticated graphical user interfaces and visualization, data quality management, and data storage (Duffy, 2016). Programmes like the UK Biobank (2016), an open resource collecting de-identified data on health and well-being from 500,000 volunteers, and making it available for research, will be instrumental in meeting these challenges. There is also the issue of obtaining informed consent about the storage and use of data, when the uses are dynamic and expanding (Khoury et al., 2016). Finally, as yet there are no centralized resources collecting datasets, modeling and software analysis tools, and pipelines for precision medicine, which would facilitate method-sharing and allow interested researchers to join the effort.

Precision approaches, as they develop, must also accommodate the ethical and transparent use of data. Recently, O'Neil has coined the phrase “Weapons of Math Destruction” (WMD) (O'Neil, 2016) to describe how black-box algorithms can create pernicious and damaging feedback loops, with unfair consequences to individuals, often without much effort placed on identifying and correcting errors (O'Neil, 2016). Hood and Friend (2011) present the vision that “in the not-too-distant future, each patient will be surrounded by a ‘virtual cloud’ of billions of data points that will uniquely define their past medical history and current health status. Furthermore, it will be possible to mine the billions of data points from hundreds of millions of individuals to generate algorithms to help predict the future clinical needs for each patient.” Hence, although precision medicine under this broad vision could have the beneficial potential to identify diseases earlier, to reduce burdens of treatment, and to improve screening by reducing false positives, and ultimately improve health, a sceptic might imagine a scenario in which these same predictions are used to produce quality-adjusted life year estimates, affecting which treatments are covered for whom, and guiding hiring, lending or health insurance decisions (O'Neil, 2016). With the amount and breadth of data available, there is the danger that such decisions could yield negative discrimination according to e.g., postcode lotteries, socio-economic factors, social network data, past healthcare interactions, judicial and law enforcement history.

How can such scenarios be avoided? Part of the answer must come from policy and regulation to ensure openness and fair use of data (Noveck, 2015). Yet, in addition, we need to develop the kind of mathematics and statistics for data science that will keep the “human in the loop” so that decision-making can be transparent and based on interpretable features and evidence. In doing so, we will need to develop methods that can track back and be updated in response to errors, taking full account of uncertainties, thus avoiding the over-reliance on complex computational decision black boxes. With this aim of model intelligibility, an important goal is to extract improved data-driven descriptions at the appropriate intermediate scales between the fully individualized level, which carries the risk of non-transparent and damaging over-use of data, and descriptions that are too coarse, which lead to insufficient precision in the face of individual variation. There is virtue in reaching a nuanced, data-informed middle-ground between these extremes: one that considers the individual in a population context and includes the role of human judgment.

It is thus essential to build theoretical understanding at the appropriate scale. One of the advantages of an integrated precision approach in medicine is to refine disease classification, increasing and finessing the number of groups of patients to reflect the true diversity of major diseases like cancers, so as to target treatment appropriately. While biomarker information can substantially improve clinical trial design as well as treatment (Trusheim et al., 2011), stratification also greatly increases the number of categories for which clinical trials may need to be carried out, reducing reproducibility and posing challenges to evidential policy (Khoury et al., 2012). Conversely, data-centric approaches may identify common mechanisms and treatments across disparate diseases, reducing stratification. Such approaches could potentially deliver dramatic cost efficiency. For these reasons, precision approaches must act at the right scale, which will often be intermediate–between “one size fits all” medicine and fully individualized therapies.

However, we do not yet have the ideal tools at hand to identify relevant features and integrate them to obtain interpretable predictions, optimized therapies, and new policies–even if merged datasets describing multivariate aspects of individuals' health across time (including, e.g., genomic, proteomic, metabolomic, brain images, social, and behavioral data) collected with informed consent were available. In addition, such combined genetic, genomic, proteomic, metabolomic, or single-cell data will only provide highly enriched and noisy snapshots taken at a few times—at best, we will have sparse noisy samples of the underlying process of disease, and sparse samples of the context of each patient.

To reveal the potential of such datasets in medicine, we must thus develop mathematical frameworks that are able to describe high-dimensional, dynamic, noisy, sparsely-sampled processes. Ideally, we must then be able to extract concise descriptions (coarse-grained at the right resolution) which are intelligible and actionable, and which link co-occurrences of events, co-morbidities, and time patterns in disease and in health-related processes. This area poses a set of core mathematical challenges: creating transparent, replicable descriptions in healthcare, which make use of large diverse datasets, placing individuals in context, and which use dynamical information across time at the correct scales. These mathematical challenges must be researched in parallel with precision medicine, ideally spanning the individual- and population-level perspectives.

In our view, these constitute deep additional challenges to mathematical modeling and data analysis that will need to be met in order for precision approaches to meet their promise. In the remainder of this perspective, we lay out a vision for what we term *precision healthcare*, its aims and its mathematical challenges. We do not aim to write a review of precision medicine; many reviews of tools and methods in different medical domains are available (see for example Chen and Snyder, 2013; Rosell and Karachaliou, 2013; Hiley et al., 2014; Ignatiadis and Dawson, 2014; Arnedos et al., 2015 among many others), as well as perspectives from a variety of viewpoints (Mirnezami et al., 2012; Roychowdhury and Chinnaiyan, 2013; Ciardiello et al., 2014; Ignatiadis and Dawson, 2014; Servant et al., 2014; Arnett and Claas, 2016; Rost et al., 2016; Vargas and Harris, 2016).

### Why precision healthcare?

For most of these challenges, population-level thinking coupled with mathematical data science analytics can help translate the benefits of precision medicine to address broader effects at the group level, including concerns regarding health equity and ethics. We use the phrase “*precision healthcare”* to encompass this vision that integrates the population and individual perspectives. Precision healthcare thus aims to build tools that make use of the increasing array of data sources, allowing for their continuous refinement in the face of new data, and whose predictions are aimed at and respond to the requirements of healthcare practitioners (clinicians, the public, policy thinkers, and other stakeholders).

This vision will require the use of an array of mathematical tools to unify individual-level precision medicine with public health, placing high-dimensional individual data and refined interventions in their social network context. Indeed, in many instances, individual health cannot be separated from its behavioral and social context. For example, highly targeted interventions against a cancer can be undermined by metabolic diseases caused by dietary behaviors which, in turn, co-vary with social network structure and other societal constructs. An adjuvant therapy for cancer might thus be to influence the diet and behavior of the patient taking into account their close social contacts.

The scenario by Hood and Friend (2011) mentioned above can thus be thought of as the analysis of a virtual cloud of a large number of high-dimensional feature vectors corresponding to the different individuals. Dynamical datasets in this scenario would correspond to a large collection of paths in such a space. If the technical and policy challenges to collect and integrate such data into a single accessible point of access were surmounted, methods for dimensionality reduction could be applied to reduce the relevant features to a few “components” which could then be used to “cluster” (or classify) the data into groups of similar individuals according to their paths. This is an area of current active research, ranging from the direct application of classic methods such as principal components analysis (PCA), support vector machines (SVMs), and independent component analysis (ICA) with all their myriad of variants, through manifold learning to the revivified use of neural networks for such classification tasks (Mallat, 2016). Developing ways to cope with noisy data and noisy labels is an ongoing challenge in machine learning (Xiao et al., 2015) and across precision medicine, as omics datasets can be extremely noisy.

However, specific requirements in the precision healthcare setting make such tasks especially difficult. The datasets are dynamic and usually sparsely sampled. The processes involved are high-dimensional, highly nonlinear, noisy, and uncertain. The dimensionality reduction framework for such datasets should ideally achieve competing objectives: preserve, to some extent, the meaning of the original descriptive variables (without mixing all features into conglomerates) while extracting concise (i.e., sparse) representations in terms of few relevant extracted features. Ideally, it should be possible to adjust the level of detail (i.e., the resolution scale) of such models depending on the quality of the data and the needs of the practitioner. Finally, the mathematical framework should deliver robust outcomes, and include the possibility of restricting and conditioning the extracted models to incorporate additional and complementary data without the need for refitting.

Indeed, in the process of harnessing these large-scale data, a great degree of caution is required. Most biomedical research is plagued by a flood of false positive results due to experiments of insufficient discriminatory power (Ioannidis, 2005). The translational impact of this trend is starkly illustrated by recent failures to reproduce landmark cancer studies and low success rates in clinical trials (Prinz et al., 2011; Begley and Ellis, 2012). In particular, the quest for (publishable) *p*-values over (meaningful) effect sizes (Goodman, 1999; Ziliak and McCloskey, 2008) has led to the likely incorrect linking of many genetic features with diseases (Johnston, 2016). Selecting appropriate mathematical models can help increase the statistical power of large-scale experimental data, allowing rigorous statistical treatments to discriminate likely from spurious effects, and quantifying the sizes of effects so that the scientific, as well as the nominally statistical, significance of observations can be better understood.

The interface of individual-level personalized medicine and public health will thus need to develop new mathematical tools to formulate and analyse mathematical questions for data-rich characterization of disease progression and transmission, controlled intervention, and healthcare provision. Key areas that we see in the remit of precision healthcare include: statistics for noisy, incomplete, heterogeneous data; stochastic modeling; inference and control of network dynamics; mathematical approaches to exploit complex structure in large datasets, and methods to couple imaging and omics. More broadly, a central distinction between precision medicine and precision healthcare is that the former treats individuals, whereas the latter treats individuals explicitly embedded in a society or broader context. Precision healthcare thus aims to link “big data” tools to explore individual agents with an understanding of how those individuals behave collectively and respond to society-wide initiatives.

### Some proposed case studies in precision healthcare

We now describe a number of demonstrative examples, ill-ustrating some of the tools that come under the umbrella of precision healthcare. These range from systems precision medicine approaches focusing on the representation of complex dynamic data, to precision healthcare approaches including both retrospective analysis and real-time interventions that are rooted in complex individual and population data.

#### Gene therapies for mitochondrial diseases

A combination of new maths, statistics, and large-scale experimental data has led to recognition of the importance of personalized therapeutic approaches in cutting-edge gene therapies addressing the inheritance of mitochondrial diseases. These diseases (e.g., mitochondrial encephalomyopathy, lactic acidosis, and stroke-like episodes—MELAS, myoclonic epilepsy with ragged red fibers—MERRF, Leber's hereditary optic neuropathy—LHON) result from mutations in mitochondrial DNA (mtDNA) which are passed from mother to child (DiMauro and Davidzon, 2005). Mitochondrial replacement therapies aim to prevent this inheritance by replacing mutated mother mtDNA with mtDNA from a third party woman, but technological limitations in the procedure can lead to small amounts of mother mtDNA being amplified leading to disease (Burgstaller et al., 2015). Classically the risk of differential proliferation has been considered minimal, but evidence harnessed with statistical modeling and large-scale data from mouse models has shown it is common (Burgstaller et al., 2014). Further, quantitative modeling on large-scale human mtDNA datasets has confirmed that this risk is present in heterogeneous human populations (Røyrvik et al., 2016), as supported by experimental observations (Hyslop et al., 2016; Yamada et al., 2016). The personalized aspect stems from the fact that the risk of differential proliferation depends on the genetic details of the mother's and third-party's mtDNA, which vary throughout global human populations according to geography and ancestry. Appropriate modeling can elucidate the biological details of why these proliferative differences arise, make probabilistic statements about the probability and timescales of therapeutic outcomes, and describe the mtDNA differences likely to arise in human populations. In the future, precision healthcare strategies could allow us to propose suitable third-party donors to optimize successful fertility strategies.

#### Pathways of disease progression in high-dimensional spaces

Recent mathematical and statistical developments in the study of evolution have shed light on the emergence of efficient photosynthesis (Williams et al., 2013) and the reduction of organelle genomes (Johnston and Williams, 2016) by modeling evolution as the acquisition (or loss) of a set of *L*-discrete traits. Evolution in this picture takes place on an *L*-dimensional hypercube, with each vertex corresponding to a given pattern of trait presence/absence and each edge corresponding to an evolutionary innovation. Observations of evolutionary intermediates can then be used, as in a hidden Markov model, to infer likely trajectories through this space. This paradigm can be developed to infer likely pathways of disease progression (generalizing statistical studies on disease progression; Hjelm et al., 2006; Pagel and Meade, 2006; Loohuis et al., 2014; Beerenwinkel et al., 2015), picturing the “space of symptoms” as an analogous hypercube, and disease progression as paths over its edges. Large-scale and longitudinal patient datasets can be used to infer likely sets of “evolutionary” trajectories through this space, so that probabilistic statements can be made about the likely next step for any given individual patient–and thus a personalized optimal therapeutic strategy. Interestingly, this approach can be linked with descriptions based on *continuous* variables, where similarity graphs are obtained from distance matrices by using graph-theoretical sparsifications that preserve the topological and geometrical structure of the data (Beguerisse-Diaz et al., 2013). The structure of the similarity graphs from the data can then be analyzed using multiscale community detection algorithms leading to highly nonlinear clustering of symptoms and individuals describing the observed pathways of disease progression (Schaub et al., 2012).

#### Social networks in health policy

Twitter provides a platform to interact directly with a large audience, and to sample and address public opinion and responses around specific issues and questions. However, it is critical to understand the different communities and conversations on Twitter, so as to target them appropriately. For example, a recent example following conversations on diabetes (Beguerisse-Díaz et al., 2017) used a unified mathematical framework (Delvenne et al., 2010; Beguerisse-Diaz et al., 2013; Beguerisse-Díaz et al., 2014; Lambiotte et al., 2015) that brings notions from stochastic processes on graphs and optimization to the analysis of Twitter networks. In this particular study, 2.5 million diabetes-related tweets were analyzed and found to fall within five broad thematic groups: health information, news, social interaction, commercial, and humor. Indeed, humorous messages and references to popular culture appear consistently, more than any other type of tweet, revealing the specific characteristics of social media interactions. The analysis of the temporal “hub” and “authority” scores of Twitter users revealed that the hub landscape is diffuse whereas the landscape of authorities is highly persistent. The Twitter authorities comprise not only bloggers, advocacy groups and NGOs related to diabetes, but also for-profit entities without specific diabetes expertise which influence the online exchanges. The top authorities fall into seven interest groups, as derived from their Twitter follower network revealing the flow of information with specific audiences. A similar analysis was carried out on the network of retweets generated by the debate surrounding the proposed adoption of the “care.data” (https://www.england.nhs.uk/ourwork/tsd/care-data/) scheme of personalized health care records by NHS England (Amor et al., 2016). In that case, a series of interest groups and conversations were identified revealing the different roles of users within and across communities, including the limited reach of some of the public policy accounts in the debate. Such findings could be used by public health professionals and policy makers to use social media as an engagement tool and to inform policy design. A similar analysis have been carried out in Beguerisse-Díaz et al. (2017) following other social movements.

#### Spreading of vaccine sentiment and spreading of vaccine preventable disease

Vaccine hesitancy and a vaccine preventable disease can be thought of as two distinct types of processes and they propagate through distinct media. We suppose that sentiment is spread socially (and is influenced by media outlets) but involves the slow evolution of beliefs rather than something as simple as the infectious propagation of a meme. In contrast, disease spread need not respect social network structure. An integrated intervention would not only target vaccination where the disease has been reported and vaccine coverage rates are low (de Figueiredo et al., 2015) but also where it is predicted to appear. Predictions would be based on integrated multi-variate “precision” data. Similarly, negative vaccine sentiment (Larson et al., 2016) could be targeted not only where it has been reported but also where it is predicted to appear given the social network structure. The coupling between belief dynamics and epidemiology now has an established theoretical presence (Wang et al., 2016) and importantly it has been observed that anti-vaccination behavior is socially clustered (Onnela et al., 2016) thereby undermining herd immunity (Salathé and Bonhoeffer, 2008); it is important to coordinate a public health response that can incorporate belief and behavior dynamics as well as the spread of infection.

#### Influencing beliefs and influencing networks

Health outcomes for chronic conditions are modulated by health behavior, which in turn might be expected to show covariation sensitive to underlying social network structure (Centola, 2010; Shalizi and Thomas, 2011; Christakis and Fowler, 2013). It has further been suggested, independent of unhealthy behavior, that social position can modulate health outcomes (Snyder-Mackler et al., 2016). There are thus a number of possible types of social interventions to improve health: (1) influencing modes of thinking to encourage critical appraisal of apparently acceptable but unhealthy behavior (changing the models that individuals use) (2) influencing health beliefs about particular topics (changing the data individuals access) (3) influencing network structure (but not social co-ordinates) to build bridges between communities for the exchange of health behavior (4) influencing the social co-ordinates of individuals (or sectors of society) and thereby altering their network neighborhood (or the gross social network structure). Changes to (3) and (4) might also affect possible physiological consequences of status comparisons (Pickett and Wilkinson, 2015). While we can cite examples of each class of intervention, these can be remarkably challenging to effect: for example showing some extreme vaccine sceptics information about the consequences of vaccine preventable disease can *increase* their vaccine scepticism (Nyhan et al., 2014); overwhelming evidence has been presented of health inequities (Marmot and Commission on Social Determinants of Health, 2007; Adler et al., 2016) but the problem persists. Challenges (1–4) constitute challenges in contemporary network science and its interface with optimal control: ideal interventions will optimally control processes on networks and optimally influence the network structure itself (Liu and Barabási, 2016).

#### Genomic epidemiology for outbreak reconstruction

**Figure 1:**
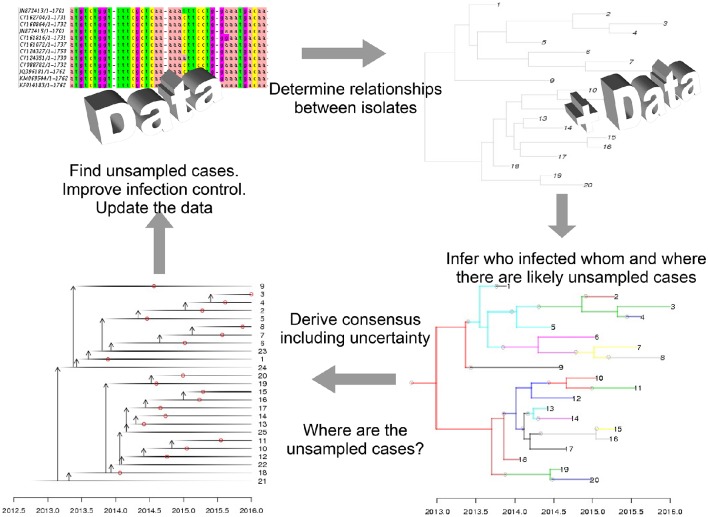
**Mathematical modeling is central to genomic epidemiology**. In precision healthcare, models that link clinical, epidemiological, and sequencing data and produce interpretable results—such as predictions for where cases have been missed—can be used to direct public health interventions.

Recent advances in sequencing technologies have driven changes in many biological domains, including epidemiology (Jombart et al., 2014; Kao et al., 2014; Colijn and Cohen, 2016). It is now feasible to obtain DNA or RNA sequences from viruses, bacteria and other pathogens, and to use these data to detect drug resistance, optimize treatments for individual patients (Vanderkooi et al., 2005; Perez et al., 2016), and to understand how pathogens are spreading and evolving by tracking small variations in the pathogen as it moves between individuals. To understand transmission, isolates are collected from patients alongside clinical data such as times of symptom onset. The isolates are sequenced and processed with bioinformatics tools, capturing even small levels of variation between patients (e.g., in a multiple sequence alignment). These can be integrated with evolutionary models to infer phylogenetic trees, describing patterns of shared ancestry among the isolates. An epidemiological model is used to define how likely a set of infection events are. This incorporates clinical information–for example, it is very unlikely that an individual would transmit an infection years before showing any symptoms, or while living in another area. Finally, mathematical models that link the phylogenetic and epidemiological information are used to compute the joint likelihood of the genetic data and the set of transmission events. This is embedded in a Bayesian approach, so the result is a posterior collection of transmission trees (who infected whom, and when), consistent with the data. There is a rapidly-growing body of work on these inference problems (Hall et al., 2015; Worby et al., 2015; De Maio et al., 2016; Klinkenberg et al., 2016; Worby et al., 2016; Didelot et al., 2017); Figure 1 is based on the approach in Didelot et al. (2017). There are natural precision healthcare applications of these tools: if more transmission is inferred to have occurred in particular locations, interventions such as improved ventilation and cleaning, early screening and active case fining can be directed there. If risk factors such as community membership, age, or co-morbidities are identified, these can be managed similarly. But perhaps the most exciting applications of these tools will happen when sequencing can be done in a matter of hours or even days. Identifying where there are likely missing cases could allow us to identify cases early, treat them, and prevent onward transmission. Real-time sequencing and infection-tracing has already had impact in the recent Ebola epidemic (Quick et al., 2016), setting the stage for this direction in public health (Gardy et al., 2015).

## Discussion

We have framed *precision healthcare* to describe the development of precision approaches which, while capturing the complexity of individual data and its societal context, extract reduced dimensionality descriptions at the relevant resolution while preserving a measure of intelligibility of the models. This can enable practitioners in the loop to use these precision approaches effectively. Such methods should be transparent, robust and able to adapt to errors and uncertainties. In bridging from the individual to the population, the methodologies should take advantage of the multivariate data sources at the heart of precision medicine, yet take the social context and population levels into account. Through several case studies in this domain of precision healthcare, we argue that this vision requires the development of new mathematical frameworks, both in modeling and in data analysis and interpretation.

Recently, “precision public health” has been characterized as delivering the right intervention to the right population at the right time (Desmond-Hellmann, 2016; Khoury et al., 2016), mirroring the oft-cited characterization of precision medicine. With support from the Gates Foundation (Cisneros, 2016), precision public health aims to apply precision (data-centered) approaches to improve the health of populations and to reduce health disparities. Public health thinkers are concerned about precision medicine's current emphasis on individual approaches, its focus on extending the use of costly genetics and other omics', and the development of tailored drug treatments (Khoury et al., 2016). Bayer and Galea report that the number of NIH projects with “public” or “population” in the title has dramatically declined, and that in 2014, research areas described with the words “genetic,” “genome,” or “gene” received 50% more funding than those with “prevention”. They are concerned that the focus on precision medicine is misguided (Bayer and Galea, 2015), and argue that improving health requires addressing persistent social realities that are not covered by access to clinical medicine (Adler et al., 2016). Persistent social inequalities can also be expected to be a major barrier in bringing advances from omics-based precision medicine to low-income countries, although recent use of rapid genomics-based tools in the Ebola outbreak (Quick et al., 2016) points to the potential to develop precision-based approaches for low-income settings.

Precision public health places emphasis on addressing such disparities, and (as with public health more generally) on prevention. In many ways, data-centered approaches have already been adopted by epidemiologists and public health practitioners and, as precision public health incorporates more individual-level data, it will require the envisaged scientific tools of *precision healthcare*. These methodologies will allow public health methods to integrate data on vaccine belief and social context with individual health records, genetic data, other biomarkers, and individual risk factors. Importantly, it is realistic to envision that the use of mobile and social network technologies will enable public health interventions typically considered at the level of populations to instead be tailored to individuals. We believe that an important aspect of the success of precision public health will depend on meeting the mathematical challenges we have outlined as precision healthcare. Identifying the right population for the right intervention will require data analysis, stratification, and modeling at the right scale: too fine, and there would be impractically many populations; too coarse, and the precision advantage is lost. It will require intelligible, transparent methods that can be communicated to public health practitioners, easily updated in the face of new data and human judgment. It will require using the right data to answer the right question, and avoiding mis-use of data to treat some populations unfairly.

It is no longer the case that the timescales of individual disease progression and the timescale of changes in health policy or social behavior are distinct. Chronic conditions from cancer to diabetes are managed over years and decades. Years and decades are equally the timescales on which other chronic problems are resolved: detrimental individual beliefs about healthy behavior, or disadvantageous social policies. The comparability of timescales of chronic diseases and chronic social problems, combined with the increase of chronic disease in the population, presents both policy, and mathematical challenges: parsimonious and predictive model choice for these slow coupled processes is an open challenge with important implications for the design of public health protocols and policies. Such problems are specific to precision healthcare: While precision medicine might integrate multiple individual-level datasets to improve treatment for a diabetic patient, it does not aim to consider the changing relevant environment and behavior (including beliefs about diet and obesity, food quality and availability, urban environments, and access to exercise). The research outlined above on social networks and health policy also exemplifies precision healthcare: it has a core set of mathematical challenges that are directly linked to healthcare (vs. medicine) and integrates opinion, engagement, delivery, and policy. While precision medicine and healthcare naturally have some overlap, the coupling of scales from individual information to societal behavior and intervention will be characteristic of precision healthcare. However, even with the best intentions, a version of precision healthcare that is highly dependent on advanced tools might be used to reduce, rather than enhance, health equity. A key challenge for precision healthcare is thus to create technologies and practices to drive us toward health equity.

## Author contributions

CC, wrote first draft. NJ, MB, IJ, SY contributed text, edited drafts, contributed to the planning stages and co-authored the paper.

### Conflict of interest statement

The authors declare that the research was conducted in the absence of any commercial or financial relationships that could be construed as a potential conflict of interest.
